# Alterations of the bile microbiome is associated with progression-free survival in pancreatic ductal adenocarcinoma patients

**DOI:** 10.1186/s12866-024-03371-9

**Published:** 2024-07-01

**Authors:** Hang Jiang, Yitong Tian, Linwei Xu, Xing Chen, Yurun Huang, Jia Wu, Tingzhang Wang, Tingting Liu, Xitian Wu, Chao Ye, Hao Wu, Wenkai Ye, Luo Fang, Yuhua Zhang

**Affiliations:** 1 Zhejiang Cancer Hospital, Hangzhou, Zhejiang 310022 China; 2 Zhejiang Chinese Medical University, Hangzhou, Zhejiang China; 3 Key Laboratory of Microbial Technology and Bioinformatics of Zhejiang Province, Hangzhou, China; 4 NMPA Key Laboratory for Testing and Risk Warning of Pharmaceutical Microbiology, Hangzhou, China; 5 Zhejiang University, Hangzhou, Zhejiang Province 310058 China

**Keywords:** Pancreatic ductal adenocarcinoma, Bile microbiome, Progression free survival

## Abstract

**Background:**

Patients with pancreatic ductal adenocarcinoma (PDAC) display an altered oral, gastrointestinal, and intra-pancreatic microbiome compared to healthy individuals. However, knowledge regarding the bile microbiome and its potential impact on progression-free survival in PDACs remains limited.

**Methods:**

Patients with PDAC (*n* = 45), including 20 matched pairs before and after surgery, and benign controls (*n* = 16) were included prospectively. The characteristics of the microbiomes of the total 81 bile were revealed by 16  S-rRNA gene sequencing. PDAC patients were divided into distinct groups based on tumor marker levels, disease staging, before and after surgery, as well as progression free survival (PFS) for further analysis. Disease diagnostic model was formulated utilizing the random forest algorithm.

**Results:**

PDAC patients harbor a unique and diverse bile microbiome (PCoA, weighted Unifrac, *p* = 0.038), and the increasing microbial diversity is correlated with dysbiosis according to key microbes and microbial functions. *Aliihoeflea* emerged as the genus displaying the most significant alteration among two groups (*p* < 0.01). Significant differences were found in beta diversity of the bile microbiome between long-term PFS and short-term PFS groups (PCoA, weighted Unifrac, *p* = 0.005). *Bacillota* and *Actinomycetota* were identified as altered phylum between two groups associated with progression-free survival in all PDAC patients. Additionally, we identified three biomarkers as the most suitable set for the random forest model, which indicated a significantly elevated likelihood of disease occurrence in the PDAC group (*p* < 0.0001). The area under the receiver operating characteristic (ROC) curve reached 80.8% with a 95% confidence interval ranging from 55.0 to 100%. Due to the scarcity of bile samples, we were unable to conduct further external verification.

**Conclusion:**

PDAC is characterized by an altered microbiome of bile ducts. Biliary dysbiosis is linked with progression-free survival in all PDACs. This study revealed the alteration of the bile microbiome in PDACs and successfully developed a diagnostic model for PDAC.

**Supplementary Information:**

The online version contains supplementary material available at 10.1186/s12866-024-03371-9.

## Introduction

Conventional wisdom says that bile is completely sterile. However, further research has identified that there is a certain amount of microbiota in bile closely relating to biliary diseases [1–3]. The homeostasis of the human microbiome is crucial for maintaining normal physiological activities, and imbalance of microbiota environment is associated with various diseases.

Pancreatic ductal adenocarcinoma (PDAC) is a malignancy of high aggressiveness, associated with a bleak prognosis and a gradually increasing incidence worldwide [4]. In addition to complex gene expression profiles, the driving factors of PDAC development involve family genetics, age, diet, lifestyle, long-term chronic inflammation [5, 6], and altered microbiome [7–9], which has received much attention in recent years. Riquelme, E et al. have demonstrated the existence of microbiota in PDAC tissue, revealing alterations in the tumor microbiome correlate with the overall survival time of PDAC patients following surgical intervention [10]. To gain a profounder comprehension of influence of the microbiome on PDAC development, Udayasuryan, B et al. conducted the microbiota transplantation experiment in nude mice confirming that microbiome could promote the progression of PDAC [11].

In addition to PDAC tissue, researchers have also found alterations of microbiota in feces [12–14], pancreatic juice [15], duodenal fluid [16], salivary [17], and bile [18]. Among the specimens that can be obtained before surgery, bile is closer to the primary pancreatic lesion than saliva, plasma, or feces, reflecting certain ignored information. During surgery, bile can also be collected for analyzing components to assist in predicting prognosis with certain research value. Compared to pancreatic juice that is only obtained during surgery, bile can be obtained before surgery in some cases, which will contribute to guiding clinical decisions to some extent avoiding certain limitations. Due to the limited sequencing data available for PDAC-related bile samples, further confirmation is needed. At the anatomical level, the bile duct is adjacent to pancreatic tissue, and the common bile duct along with pancreatic duct merge into the duodenum. Due to their close anatomical relationship, alterations may occur in the bile microbiome of PDAC patients.

In present study, we gathered bile samples from 45 PDACs, including 20 matched pairs before and after surgery, and 16 patients with benign biliary disease who underwent surgery for 16srRNA sequencing to explore whether the bile microbiome of PDAC patients has changed. We also analyzed whether changes in the bile microbiome of PDAC patients correlated with tumor marker levels, disease staging, before or after surgery. Furthermore, PDAC patients were divided into long-term progression free survival (PFS) and short-term PFS groups, to assess variations in the composition of bile microbiota among two groups. Finally, we first constructed a prediction model based on bile biomarkers, aiming to use it as a novel diagnostic tool.

## Methods and materials

### Patient enrollment and biospecimen collection

For this study, forty-five patients diagnosed with PDAC, and sixteen benign controls were enrolled during a routine surgical visit at Zhejiang Cancer Hospital in China. Participants aged between 18 and 75 years old were eligible. However, Individuals were excluded from the study if they had any of the following conditions: (a) a concurrent diagnosis of another type of malignant tumor, (b) a past history of myocardial infarction or any other cardiovascular diseases, (c) a record of organ transplantation or immune system-related illnesses, (d) Significant mental health issues, (e) a history of substance abuse, or (f) were currently pregnant or in the perinatal period. Additionally, (g) patients were required not to have undergone antibiotic treatment within six months prior to biospecimen collection, as this period is generally considered sufficient for the microbiome to recover following antibiotic administration.

Routine use of Percutaneous transhepatic cholangial drainage (PTCD) puncture for jaundice reduction is conducted on patients with preoperative obstructive jaundice. For other patients, bile is obtained from the surgical specimens. One week after surgery, bile is obtained through preoperative placement of PTCD tube or in-surgery placement of T-tube.

A total of 61 patients fulfilled the requirements for inclusion in the analysis, comprising 45 patients with PDAC and 16 control individuals. Table 1 presents a comprehensive description of the patients. Prior to their participation in the study, all individuals provided written informed consent. The research adhered strictly to the ethical guidelines established in the 1975 Helsinki Declaration.

### DNA extraction and sequencing of 16 s rRNA amplicon

Using the DNA Isolation Kit, DNA was extracted from bile, strictly adhering to the manufacturer’s instructions. The concentration and purity of the extracted DNA were visually evaluated on 1% agarose gels. Following this inspection, the DNA was diluted to a concentration of 1ng/µL with sterile water. Amplification of the bacterial 16 S rRNA V3 region was achieved using a universal primer pair, and the PCR reaction was carried out with Phusion® High-Fidelity PCR Master Mix. Following agarose gel electrophoresis of the PCR products, DNA fragments measuring approximately 400 to 450 bp were chosen for further manipulation. The TruSeq® DNA PCR-Free Sample Preparation Kit from Illumina was employed to create libraries, following the manufacturer’s prescribed steps. Thereafter, sequencing of these libraries took place on the Illumina MiSeq platform.

### Clustering and annotation of OTU, analysis of bacterial diversity and functional annotation using KEGG pathway

The 300 bp paired end reads underwent assembly utilizing FLASH (V1.2.11), followed by quality filtering with QIIME (V1.9.1). Using UCHIME, chimera sequences were eliminated, and subsequent sequence analysis was performed with Uparse software (V8.1.1861). Operational taxonomic units (OTUs) were assigned to the sequences, with a similarity threshold of 97% or higher guiding the grouping process. For OTU annotation, the SILVA reference database was utilized.

To assess the complexity of the microbial diversity within each sample, alpha diversity analysis was performed employing six indices: Observed-species, Chao1, Shannon, Simpson, ACE, and Good-coverage. These indices were calculated using QIIME (Version 1.9.1) and were subsequently visualized through R software (Version 3.2.2).

Beta diversity analysis was conducted to evaluate the variations in the complexity of microbial composition across diverse samples. Beta diversity was measured by employing both weighted and unweighted UniFrac metrics, which were calculated using QIIME software (V1.9.1). Before performing cluster analysis, the dimensionality of the original variables was reduced through a principal component analysis (PCA) using the FactoMineR and ggplot2 packages in R software (V3.2.2). After conducting PCA, Principal Coordinate Analysis (PCoA) was utilized to extract principal coordinates, facilitating the visualization of intricate, multidimensional data. This involved transforming the distance matrix of weighted or unweighted UniFrac among samples into a collection of orthogonal axes. In this visualization, each principal coordinate captured a decreasing order of variation factors. The results of the PCoA analysis were presented using the vegan, stat, and ggplot2 packages in R software (V3.3.0). Performed the T-test on the relative abundance of samples among groups to identify significant differences (*p* ≤ 0.05).

To identify statistically significant biomarkers, the Linear discriminant analysis effect size (LEfSe) approach was employed through an online tool, enabling the screening of differentially abundant features [19]. The functional characteristics of the microbial community was predicted utilizing the Tax4Fun software [20].

### Constructing a PDAC diagnostic model based on bile microbiome

To assess significant differences in OTUs between two groups, Student’s t-test was utilized. Subsequently, a random forest classification model was employed with five-fold cross-validation on the filtered OTUs. Utilizing cross-validation results, a preeminent trio of biomarkers was selected for assessing the model’s capacity to predict disease occurrence. The receiver operating characteristic (ROC) curve was plotted leveraging the pROC package, with the area under the ROC (AUC) curve serving as a metric to gauge the model’s predictive performance.

## Results

### Alteration of bile microbiota diversity in PDAC

In the current research, a total of 81 bile samples were gathered from the department of Hepatobiliary and pancreatic surgery at Zhejiang Cancer Hospital. Among them, 65 belonged to PDAC patients, of which 20 pairs were paired before and after surgery, the remaining 25 were unpaired PDAC bile samples. 16 benign control bile samples were collected from patients with benign biliary diseases, with leading indications in controls were bile duct stones and gallbladder polyps. Detailed clinical information regarding the patients has been incorporated in Table 1.

**Fig. 1:**
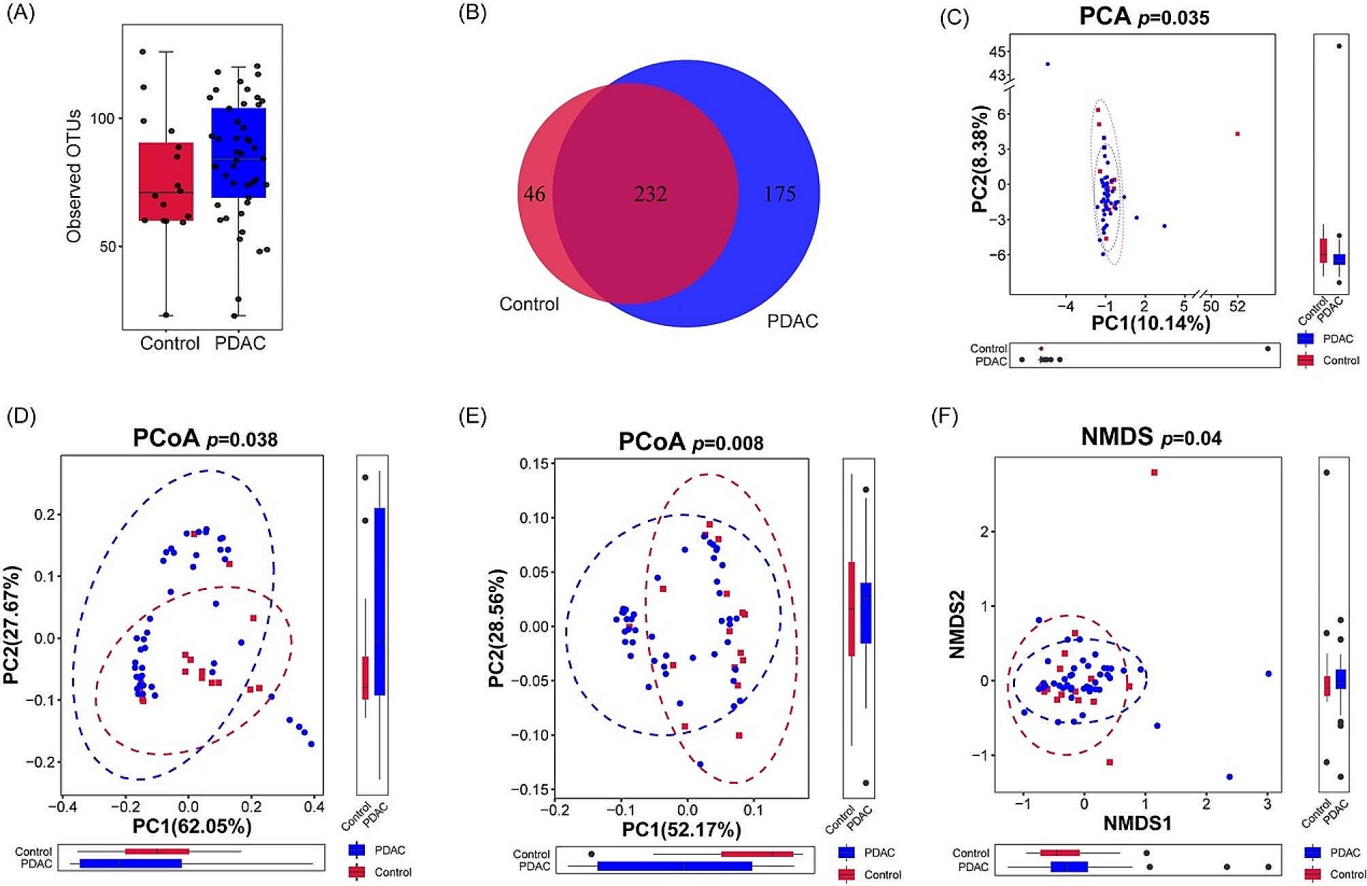
Biodiversity and Compositional Profile of Bile Microbiota. (**A**) Observed OTUs in both groups. (**B**) Venn Diagram of Overlaps in Bile Microbiota Composition Based on OTUs. Beta-diversity of bile microbiome based on PCA, R²=0.0422 (**C**), PCoA Weighted Unifrac distance, R²=0.0475 (**D**), PCoA Unweighted Unifrac distance, R²=0.0429 (**E**), NMDS, R²=0.0422 (**F**). OTU, operational taxonomic unit; PCA, principal component analysis; PCoA, principal coordinate analysis; NMDS, nonmetric multidimensional scaling

The OTUs of PDAC (*n* = 45) and benign controls (*n* = 16) were first identified. Although the difference in the median values of OTU number existed among two groups, there is no significant difference in the OTU number (Fig. 1A). Furthermore, a Venn diagram was utilized to elaborate the relationship between the two groups. Among the 453 total OTUs identified, 221 were commonly observed in both groups, whereas 175 were exclusively present in the PDAC group and 46 were uniquely found in the benign control group. (Fig. 1B).

To further comprehend the alteration in the bile microbiome, we analyzed Alpha diversity indices such as Ace, Shannon, and Simpson. Nevertheless, no notable differences were identified in the alpha diversity of the bile microbiome among two groups.

Beta diversity was visualized through NMDS, PCA, and PCoA using Weighted Unifrac distance and Unweighted Unifrac distance (Fig. 1C-F), to enhance our comprehension of bile microbiome diversity and its relationship to disease. Significant differences were found in beta diversity of the bile microbiome between PDAC and benign control (PERMANOVA omnibus test, *p* < 0.05).

These results indicated that significant differences existed in the biodiversity of bile microbiome between the PDAC and benign control groups, necessitating further analysis to gain a deeper comprehension.

**Table 1:** Clinico-pathological Characteristics of PDAC and benign control Patients

Factor	PDAC(*n* = 45)	Benign control(*n* = 16)
Age(years)	69(34–80)	53(33–72)
Gender(F/n)	18/45	7/16
BMI Hypertension(n) Diabetes(n) Jaundice Tumor location	21.8 ± 2.8 18(40.0%) 11(24.4%) 19(42.2%)	24.56 ± 3.0 3(18.8%) 2(12.5%) 0(0.0%)
Pancreatic head Pancreatic head and neck	44 1	
TNM staging (n)		
I II III IV	8(17.8%) 21(46.7%) 14(31.1%) 2(4.4%)	
CA199 at diagnosis (u/mL)		
≤37/>37	11/34	

Data are presented as mean ± SD or median (1st-3rd quartile) depending on the normality of the distribution. F: female, PDAC: pancreatic ductal adenocarcinoma, BMI: Body Mass Index

### PDACs bile harbor a unique and diverse microbiome

With knowing the alteration of bile microbiome biodiversity in PDACs, we proceeded to identify the specific constituents of the bile microbiota, focusing on its abundance.

**Fig. 2:**
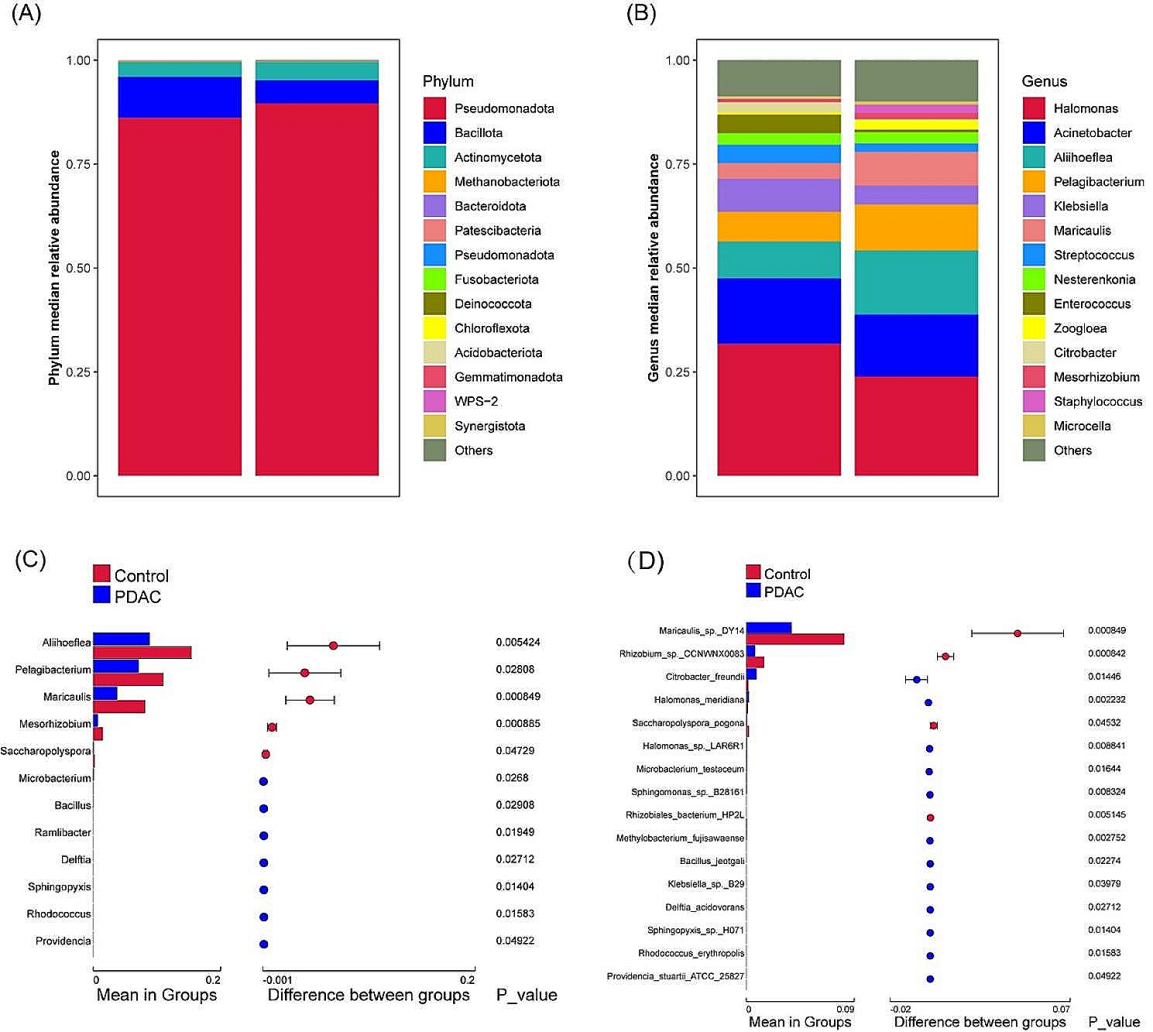
Bile microbiota composition in PDAC and benign control. (**A**) Median relative abundances at the phylum level, and (**B**) median relative abundances at the genus level. The 14 most abundant genera are represented, while all others are grouped under the category ‘others’. (**C**) Significant differences in genera between the two groups. (**D**) Significant differences in species between the two groups

Due to the lack of clarity regarding the fundamental Composition of the bile microbiome in a healthy state, we conducted an investigation into the bile core microbiome profiles of both benign controls and PDAC patients at the phylum taxonomic level (Fig. 2A). *Pseudomonadota* was the most abundant phylum, comprising 86.1% of total OTUs in PDACs and 89.6% in benign controls, respectively. *Bacillota* and *Actinomycetota* followed closely, with these three phyla collectively accounting for over 90% of the total OTUs. No significant differences were found in phylum of the bile microbiome composition between PDAC and benign control, but the significant differences of other levels were identified (Supplementary Figure S1).

We conducted a deeper analysis of the genus-level composition of bile microbiota (Fig. 2B). Notably, *Halomonas* was the dominant genus in both groups, followed closely by *Acinetobacter*, *Aliihoeflea*, and *Pelagibacterium*. Furthermore, we revealed significant differences in the abundance of 12 genera between the two groups. Interestingly, only five genera—*Aliihoeflea*, *Pelagibacterium*, *Maricaulis*, *Mesorhizobium*, and *Saccharopolyspora* were observed to be enriched in the benign controls, while the remaining genera exhibited enrichment in PDACs. (Fig. 2C).

In the genus level, we identified differences in bile microbial community composition between PDACs and benign controls. To better determine whether there is a single bacterial species with differences, we conducted further analysis at the species taxonomic level (Fig. 2D). Among the species identified, 16 species were found to differ significantly between the two groups. Notably, only four species—*Maricaulis_sp._DY14*, *Rhizobium_sp._CC-NWNX0083*, *Saccharopolysporapogona*, and *Rhizobiales_bacterium_HP2L*—were observed to be enriched in the benign controls, while the remaining species exhibited enrichment in PDACs.

### Essential bile microbes and microbial functions in relation to PDAC

**Fig. 3:**
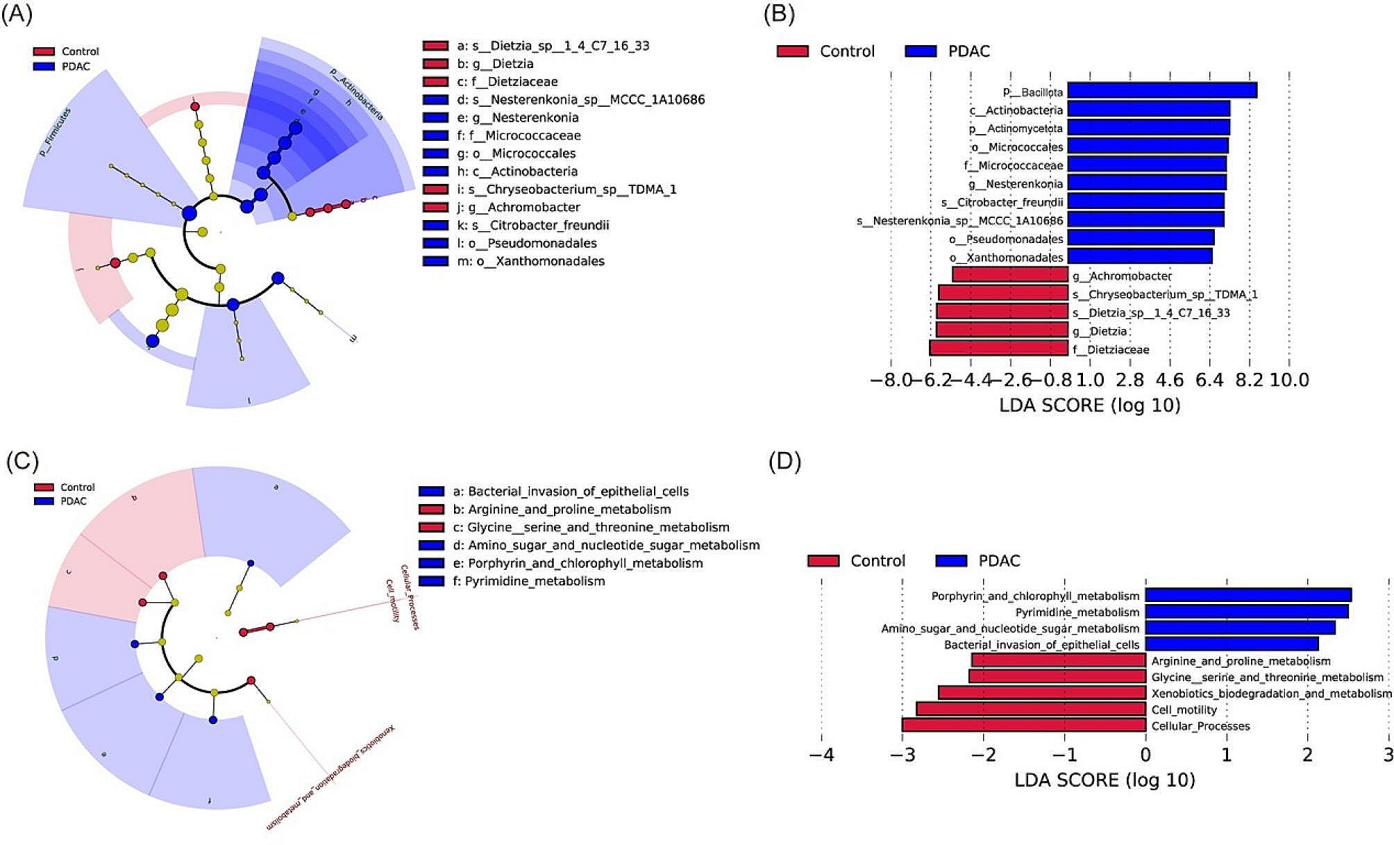
Identification of bile microbial biomarkers and microbial community functions in two groups using LEfSe analysis. (**A**) Cladogram representing specific microbial taxa in both groups. (**B**) Histogram displaying LDA scores for differentially abundant microbial biomarkers between the two groups. (**C**) Cladogram depicting community function profiles of both groups. (**D**) Histogram showing LDA scores for differentially enriched community functions between the two groups. LEfSe, linear discriminant analysis effect size; LDA, linear discriminant analysis

LEfSe analysis was performed to assess the vital microbial biomarkers that distinguish between PDAC and benign control groups. As depicted in Fig. 3A, B, two phyla *Bacillota* and *Actinomycetota*, one class *Actinomycetota*, three orders *Micrococcales*, *Pseudomonadates* and *Xanthomonadates*, one familiy *Micrococcaceae*, one genus *Nesterenkonia*, and two species *Citrobacter_freundii* and *Nesterenkonia_sp_MCCC_1A10686* were notably enriched in PDACs. one family *Dietziaceae* was found enriched in benign controls, as well as two genera including *Achromobacter* and *Dietzia*. Beside two species *Dietzia_sp_14C71633* and *Chrysepbacterium_sp_TDMA_1* were also increased in benign controls.

Furthermore, we predicted the functional profiles of the bile microbial community by utilizing the KEGG pathway and KEGG orthology (Fig. 3C, D). Four distinct pathways were identified in the PDAC group, including Amino sugar and nucleotide sugar metabolism, Bacterial invasion of epithelial cells, Pyrimidine metabolism as well as Porphyrin and chlorophyll metabolism. Five specific pathways were identified in the benign control group, including Glycine serine and threonine metabolism, Cell motility and Cellular Processes, Arginine and proline metabolism as well as Xenobiotics biodegradation and metabolism.

### Microbe abundance correlates to CA199 levels in PDACs

CA199 servers a serum biomarker playing a crucial role in diagnosing PDAC, with its levels closely linked to disease prognosis. Based on serum CA199 levels, 45 PDAC patients were divided into two groups: 20 patients with high serum CA199 levels (> 300U/mL) (CA199-H) and 25 patients on the contrary (CA199-L). The same analyzes were conducted to observe any alteration in the bile microbiome between both groups of patients.

The Venn diagram demonstrated that 179 OTUs were common to both groups among the total 381 OTUs, whereas 74 and 105 OTUs were exclusively present in each group, respectively. (Supplementary Figure S2A). To delve deeper, we explored the relative abundance of bile microbiota across various taxonomic ranks. Specifically, at the order level, *Oligoflexales* demonstrated a noteworthy distinction between the two groups, displaying greater abundance in the CA199-L group. Similar result was displayed in Family level with *0319-6G20*. In the species level, we further identified disparities in the composition of the bile microbial community between the two groups and *Nesterenkonia_sp._DL29* were observed enriched in CA199-L group (Supplementary Figure S2B-D).

**Fig. 4:**
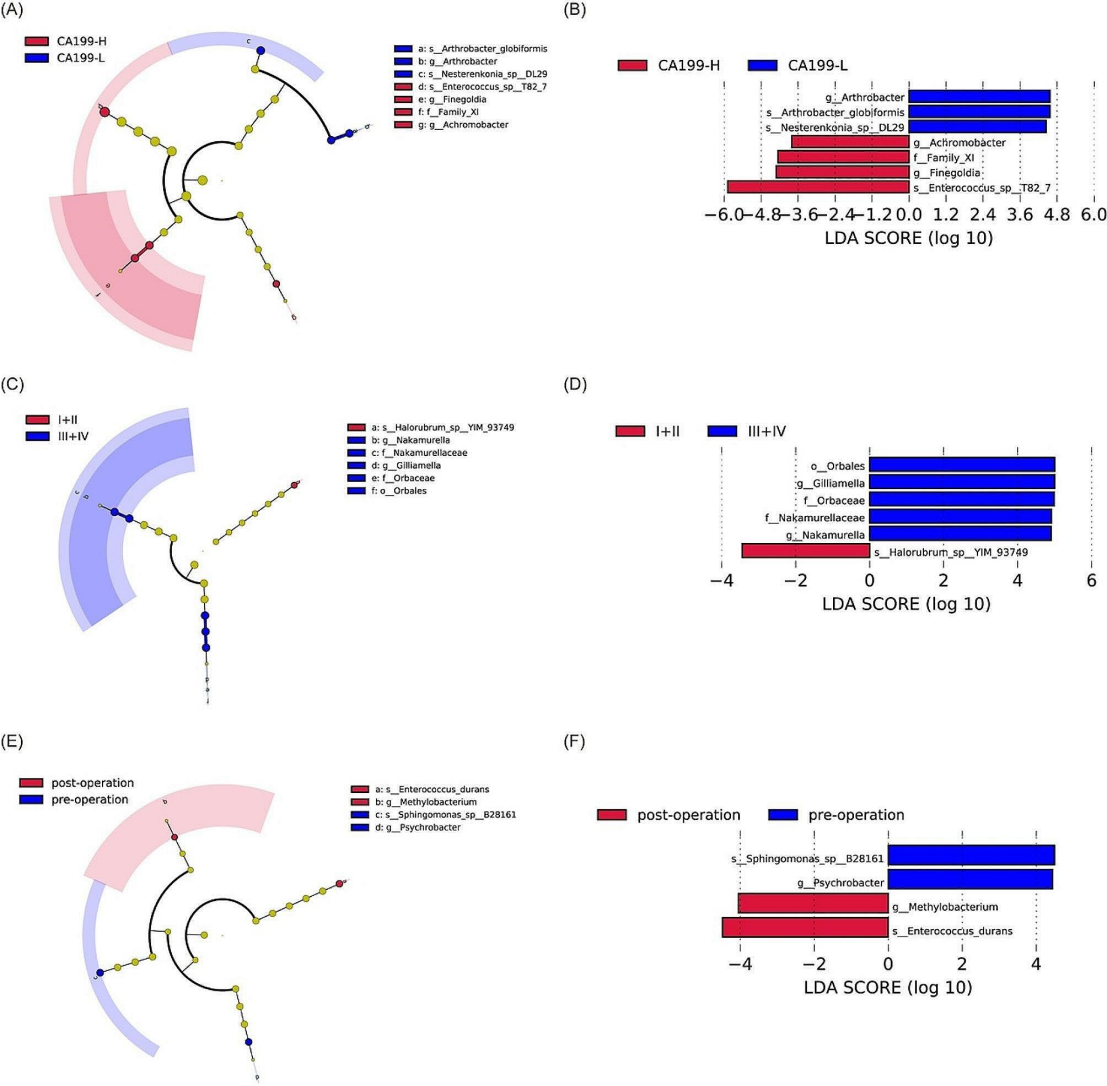
Identification of bile microbial biomarkers in diverse groups using LEfSe analysis. (**A**, **B**) CA199-H and CA199-L groups. (**C**, **D**) I + II and III + IV groups. (**E**, **F**) post-operation and pre-operation groups

With knowing the different bile microbiota abundance in two groups, we next analyzed the crucial microbial biomarkers between them (Fig. 4A, B). One genus *Arthrobacter* and two species *Arthrobacter_globiformis* and *Nesterenkonia-sp._DL29* were significantly enriched in CA19-9-L group. One family *Family XI*, two genera *Achromobacter* and *Finegoldia*, as well as one specie *Enterococcus_sp_T82_7* was increased in CA199-H group.

### Microbe abundance correlates to the course of disease in PDACs

After confirming the difference in the bile microbiome composition between PDAC group and benign control group, as well as between the CA199-H and CA199-L groups, we proceeded to investigate whether the PDAC bile microbiome is associated with disease staging. According to disease staging 45 patients with PDACs were divided into three groups: 29 patients in I + II, 16 patients in III + IV. The same analyzes were conducted to observe any differences in the bile microbiota between these two groups of patients.

As depicted in the Venn diagram (Supplementary Figure S3A), 187 OTUs out of the total 382 were commonly shared by both groups, while 138 and 49 OTUs were exclusively present in the I + II and III + IV groups, respectively. Subsequently, we conducted a further analysis to explore the relative abundance of bile microbiota across various taxonomic levels. (Supplementary Figure S3B-F)

Crucial microbial biomarkers between them were further identified (Fig. 4C, D). In III + IV group, *Orbalesand* was increased in order level. *Orbaceae* and *Nakamurellaceae* were enriched in family level. *Gilliamella* and *Nakamurella* were identified as biomarkers in genus level. In I + II group, *Halorubrum-sp_YIM_93749* was identified as a biomarker.

### Altered bile microbiome in PDACs after operation

Comparing bile samples from the same patient before and after surgery, we attempted to assess whether surgery alters the composition of PDACs’ bile microbiome. There were 20 patients with PDACs who had matched preoperative and postoperative bile samples (Supplementary Figure S4). *Allorhizobium-Neorhizobium-Pararhizobium-Rhizobium* was identified as a significantly different genus between preoperative and postoperative group. With aspect of crucial microbial biomarkers, *Psychrobacter* and *Sphingomonas_sp_B28161* served as genus level and species level biomarker in preoperative group. *Methylobacterium* and *Enterococcus_durans* were identified as genus level and species level biomarker in postoperative group (Fig. 4E, F).

### Patients with long-term progression-free survival harbored a unique and diverse bile microbiome

We further explored whether there would be different bile microbiomes between patients with long-term PFS and short-term PFS in PDACs. We defined the short-term PFS as less than 180 days and the long-term PFS as more than 360 days. Among them, 13 patients had a short progression-free survival, and 19 patients on the contrary.

**Fig. 5:**
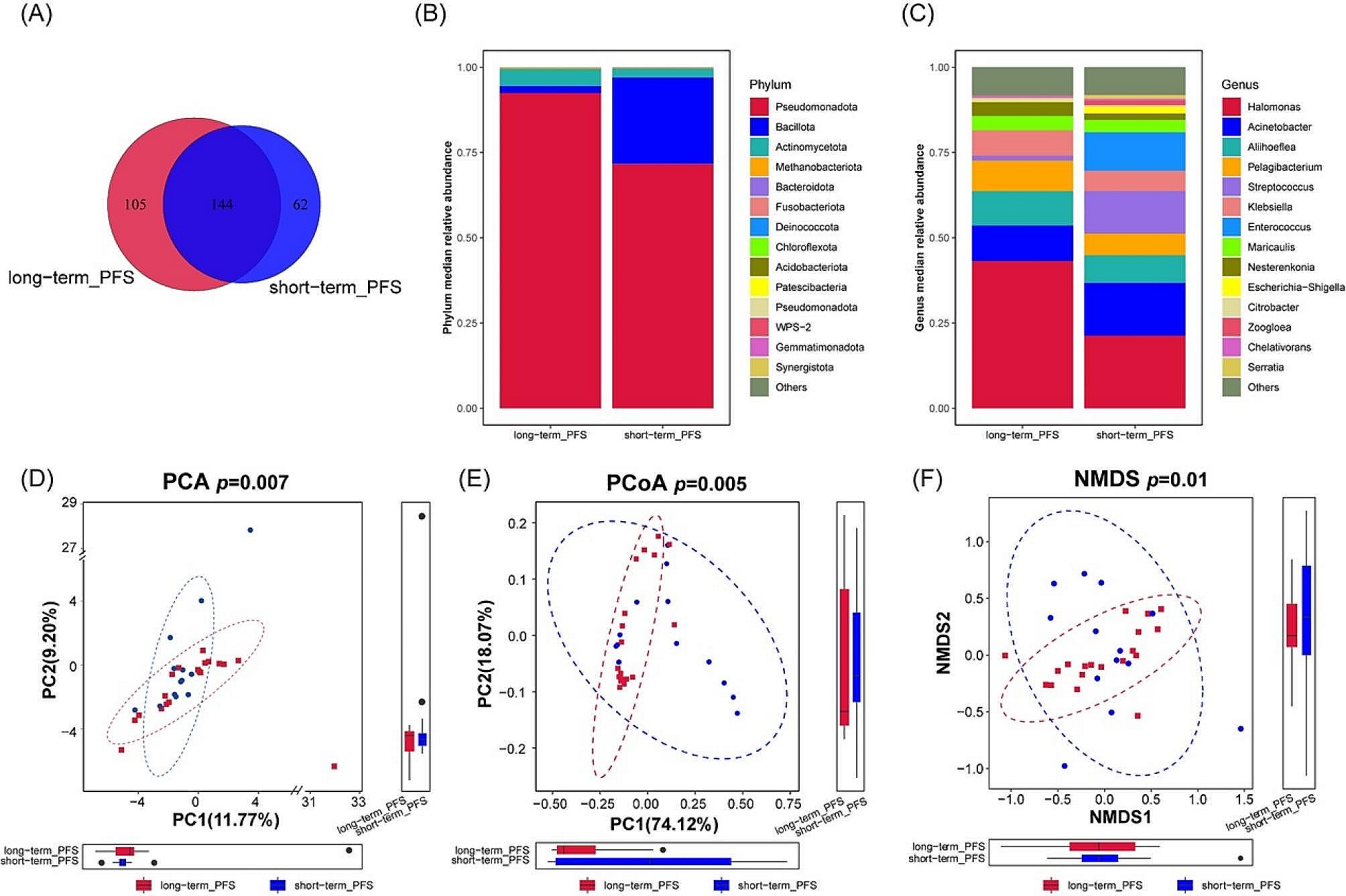
Bile microbiota composition in short-term PFS and long-term PFS patients. (**A**) Venn Diagram Illustrating Overlapping Components of Bile Microbiota Composition Based on OTUs. (**B**) Median relative abundances at the phylum level, and (**C**) median relative abundances at the genus level, with the top 14 most abundant genera represented and all remaining genera grouped under the category ‘others’. Beta-diversity of bile microbial community based on PCA, R²=0.0972 (**D**), PCoA, R²=0.1491 (**E**), NMDS. R²=0.0972 (**F**). PCA, principal component analysis; PCoA, principal coordinate analysis; NMDS, nonmetric multidimensional scaling

As indicated in the Venn diagram (Fig. 5A), 167 OTUs out of the total 311 were commonly shared between the two groups, while 105 and 62 unique OTUs were uniquely possessed by the long-term PFS and short-term PFS groups, respectively. We further investigated the biliary core microbial profiles between two groups on the phylum and genus taxonomic level (Fig. 5B, C). Beta diversity was visualized by NMDS, PCA, and PCoA using Weighted Unifrac distance (Fig. 5D-F). Significant differences were found in beta diversity of the bile microbiome between long-term PFS and short-term PFS groups (PERMANOVA omnibus test, *p* < 0.05).

**Fig. 6:**
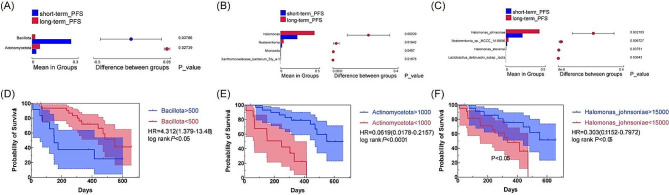
Identification of differential bacteria between short-term PFS and long-term PFS groups (**A**) Distinct Phyla, (**B**) Genera, (**C**) Species Identified between two Groups. Survival analysis of the relationship between Bacillota (**D**), Actinomycetota (**E**), Halomonas_johnsoniae (**F**) and PFS of PDAC patients

Further investigation was conducted to explore the relative abundance of bile microbiota across various taxonomic levels. (Supplementary Figure S5). In phylum level, *Bacillota* enriched in short-term PFS group and *Actinomycetota* enriched in long-term PFS group (Fig. 6A). In genus level, long-term PFS group harbored four genera with significant difference between two groups as well as four species (Fig. 6B, C). To further explore whether the expression levels of differential bacteria have prognostic significance in all PDAC patients, we divided the patients into various groups based on the expression levels of the differential bacteria. When the expression level of *Bacillota* in bile was above 500, the patient’s progression-free survival was shorter (Fig. 6D, *p* < 0.05). Conversely, with the expression level of *Actinomycetota* exceeding 1000, the outcome for patients was better (Fig. 6E, *p* < 0.0001). Focusing on the differentially expressed strain *Halomonas_johnsoniae*, when its expression abundance in PDAC bile was more than 15,000, the patient’s progression-free survival was longer (Fig. 6F, *p* < 0.05).

With knowing the different bile microbiota abundance in two groups, we next analyzed the crucial microbial biomarkers between them (Supplementary Figure S6). One family *Devosiaceae*, three genera *Zoogloea*, *Acidovorax* and *Ramlibacter*, as well as one species were identified as biomarkers in long-term PFS group.

### Exploring the diagnostic potential of PDAC through bile microbial biomarkers

**Fig. 7:**
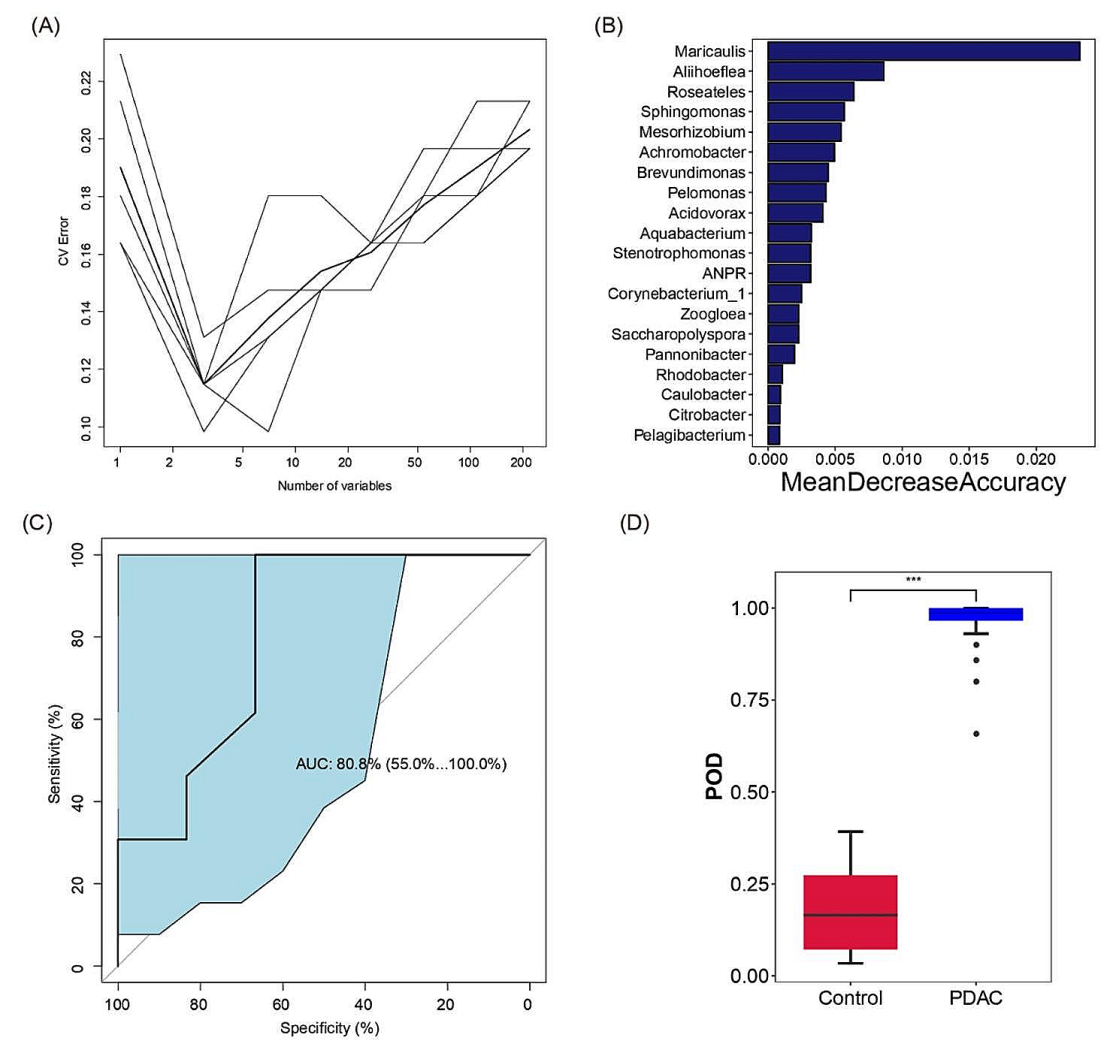
Identification of PDAC Diagnostic Biomarkers in Bile Microbiota Using OTU-Based Random Forest Model. (**A**) Fivefold cross-validation was conducted with varying numbers of OTUs. (**B**) Listed are the top 20 tested OTUs, with a higher MeanDecreaseAccuracy value indicating greater significance in the classification model. (**C**) The area under the curve (AUC) between the two groups was 80.08% (95% CI: 55.0–100%). (**D**) The probability of disease (POD) value was significantly different between PDACs and benign controls, ***p < 0.001. AUC, area under the curve; CI, confidence interval; POD, probability of disease

Microbial biomarkers exhibit the close correlation with certain human diseases [21]. Developing a prediction model to distinguish PDACs from benign controls based on bile microbial biomarkers is an intriguing pursuit, offering a promising method for clinical diagnosis of PDAC. To achieve this, we leveraged samples from the PDAC group to construct a random forest classification model. Through rigorous testing involving various numbers of operational taxonomic units (OTUs), we identified a set of three OTUs as the optimal biomarker combination, validated through fivefold cross-validation (Fig. 7A). Figure 7B presented the top 20 OTUs along with their MeanDecreaseAccuracy values, providing a comprehensive overview of the most influential microbial markers. Notably, the area under the ROC curve (AUC) achieved an impressive 80.8%, with a 95% confidence interval ranging from 55.0 to 100% (Fig. 7C), indicating the high diagnostic accuracy of our model. Additionally, Fig. 7D clearly demonstrates a substantial increase in the probability of disease (POD) value in the PDAC group compared to the benign control group, achieving a significant *p*-value of less than 0.0001, further strengthening the diagnostic potential of our model. However, the validation of this model was hindered by the limited sample size of this study, as well as the currently limited data regarding the bile microbiome of PDAC patients.

## Discussion

Overall, the development of PDAC is influenced by numerous factors, and the impact of the microbiome cannot particularly be ignored. In human body, the microbiome is a complex and dynamically changing micro-ecosystem closely relating to individual life activities [22]. For PDAC, the difference in microbiota communities between the tumor and normal tissue has been identified associating with survival time [10]. Among the PDAC available samples, researchers also analyzed the alterations in the microbiome of saliva, feces, plasma, pancreatic juice, duodenal juice, and bile. Among the pre-operative samples available, bile may reflect information with concealing in routine samples due to its proximity to the anatomical relationship with the primary pancreatic lesion and obtaining bile specimens before surgery is feasible with certain research value. With further research, it has been identified that there is a certain amount of microbiota in bile closely relating to biliary disease. Anatomically, the common bile duct and pancreatic duct merge into the duodenum, it is possible that the biliary microbiota of pancreatic cancer patients has also changed. Although previous studies have involved the analysis of biliary microbiota in PDAC patients [15, 18, 23], the results still need to be further confirmed due to the limited size of the bile sample and the restricted sequencing data available for PDAC-associated bile samples.

In our study, we gathered bile samples from patients diagnosed with PDAC and benign biliary diseases at the Zhejiang Cancer Hospital. Subsequently, the comprehensive analysis of the bile microbiota community composition was conducted using 16 S rRNA amplicon sequencing. We used sequencing to reveal the composition of bile microbiome in PDAC, the alterations of which correlated to CA199 level, disease staging, surgery and PFS. When compared to benign controls, the biodiversity of the bile microbiota has been identified as significantly altering in the PDAC group. Furthermore, through the application of a random forest classification model, we made the initial discovery of biomarkers for PDAC diagnosis. These outcomes hinted at the potential of biomarkers from the bile microbiota as a novel diagnostic tool for PDAC. The stability of the microbiota is crucial for maintaining normal physiological processes, and the disruption of microbiota homeostasis is closely associated with disease progression [24]. The upregulation of microbiota diversity in PDACs, to some extent, that the growth of microbiota existing in the normal state is inhibited.

In phylum level, the abundance of *Bacillota* was slightly altered in PDACs compared to benign controls. Correspondingly, A notable variation in the abundance of *Bacillota* was observed between the short-term PFS group and the long-term PFS group, which correlated with the overall PFS in PDAC patients. *Bacillota* play a role in the dehydration of bile acids, transforming them into deoxycholic acid and lithocholic acid, both detrimental to cell [25]. *Actinomycetota* are enriched in long-term PFSs and, converse to *Bacillota*, are associated with overall PFS in PDACs. At the genus level, 12 genera were identified as being differentially enriched in PDAC and benign control groups, with five of the highly abundant genera being concentrated in the benign control group, indicating that the abundance of dominant microbiota communities in bile has changed, affecting the bile microbiome homeostasis. Compared to the short-term PFS group, four genera were identified as being enriched in the long-term PFS group. Specifically, *Halomonas_johnsoniae* was enriched in the long-term PFS group at the species level, and its abundance was found to be associated with patient prognosis. We further constructed a PDAC prediction model based on bile microbial biomarkers, but due to the scarcity of bile samples, the limited sample size and current research constraints, the model needs further validation.

Our analysis of the functional characteristics of the bile microbial community revealed distinct enrichments in several pathways between the two groups. Specifically, four pathways were enriched in the PDAC group: Bacterial invasion of epithelial cells, Amino sugar and nucleotide sugar metabolism, Pyrimidine metabolism as well as Porphyrin and chlorophyll metabolism. Pyrimidine metabolism is a hallmark of cancer and is associated with resistance to gemcitabine in PDAC [26]. Modulating Pyrimidine metabolism can slow down the disease progression to some extent. Bacterial invasion of epithelial cells can exacerbate the disease progression by regulating cell proliferation, migration, and invasion [27, 28]. In PDAC, abnormal tumor metabolism plays a promotive role in disease progression. Distinct groups based on tumor marker levels, disease staging, and before and after surgery did not identify differences in the microbiota. This may be due to the limited sample size, and the bile microbiome in PDAC requires further investigation.

As bile microbiome research is still in its infancy, we analyzed the original sequencing data of special bile samples to restore their original appearance without deleting the so-called kitome taxa [29]. This is because removing kitome sequences from special samples, as reported by peers, may make it difficult to obtain unique microbiome information from those samples [30]. The microbiome of bile samples is not entirely the same as that of other parts of the human body. Moreover, some species in the kitome taxa naturally exist in the human body and are closely related to the occurrence and development of diseases [10, 31–36]. Deleting kitome sequences prematurely may lead to the loss of potentially important information. Since we did not delete the kitome sequences, we made every effort to minimize the possibility of contamination both pre- and post-sequencing. Firstly, during the sample collection phase, we thoroughly implemented aseptic principles and properly preserved the samples. Secondly, in the experimental process, we used Water nuclease-free as a negative control and performed extraction, library construction, and sequencing experiments synchronously with other samples to ensure that experimental operations did not affect the sample data. Finally, we overlapped the kitome data with our sequencing data for reference (Supplementary Table S1). Due to the special nature of bile samples and their unique microbiome composition, we did not delete the kitome taxa. Although the kitome may indicate potential contamination of the data, given the specificity of bile samples, we still retained them. Some of these bacteria are closely related to disease progression, and we retained this information to explore its potential value. Kitome data may cause misinterpretation of experimental results, but to ensure the authenticity of sample sequencing and avoid losing potentially important information, we made every effort to minimize the possibility of contamination and followed the practices of our peers to retain and analyze these data [1, 37–39].

Here, we revealed compositional differences in the bile microbiome between PDAC and benign control groups. We discovered a combination of bile microbial biomarkers capable of distinguishing PDAC patients from those with benign conditions, achieving a relatively high specificity of 80.8%. Nevertheless, the limited sample size precluded the conduct of a validation study in the PDAC patient cohort. Consequently, larger-scale studies are imperative to further assess the clinical utility of the established classifier.

## Conclusions

In conclusion, our study has identified compositional differences in the bile microbiome between PDAC and benign control groups. With in-depth group analysis, we found differential bacteria between the long-term and short-term PFS groups being associated with PFS in all PDAC patients. Bile bacterial species have the potential to serve as valuable biomarkers for diagnosing PDAC and distinguishing it from benign conditions. We first constructed a PDAC prediction model based on bile microbiome as a diagnostic tool. Overall, these results suggest that the distinct mechanisms and impacts of the biliary microbiota in PDAC patients merit further investigation.

### Electronic supplementary material

Below is the link to the electronic supplementary material.

Supplementary Material 1

Supplementary Material 2

## Data Availability

Sequences obtained were deposited in National Genomics Data Center (NGDC), China National Center for Bioinformation (CNCB) with project numbers PRJCA024210 and Genome sequence archive (GSA) CRA015298. (https://ngdc.cncb.ac.cn/bioproject/browse/PRJCA024210)

## Abbreviations

PDAC: pancreatic ductal adenocarcinoma
PFS: progression free survival
PTCD: Percutaneous transhepatic cholangial drainage
OUT: operational taxonomic unit
KEGG: Kyoto Encyclopedia of Genes and Genomes
PCA: principal component analysis
PCoA: Principal Coordinate Analysis
LEfSe: Linear discriminant analysis effect size
ROC: receiver operating characteristic
AUC: area under the ROC
LDA: linear discriminant analysis
POD: probability of disease
CI: confidence interval

## References

[1] Liwinski T, Zenouzi R, John C, Ehlken H, Rühlemann MC, Bang C (2020). Alterations of the bile microbiome in primary sclerosing cholangitis. Gut.

[2] Sabino J, Vieira-Silva S, Machiels K, Joossens M, Falony G, Ballet V (2016). Primary sclerosing cholangitis is characterised by intestinal dysbiosis independent from IBD. Gut.

[3] Jia X, Lu S, Zeng Z, Liu Q, Dong Z, Chen Y (2020). Characterization of gut microbiota, bile acid metabolism, and cytokines in Intrahepatic Cholangiocarcinoma. Hepatology.

[4] Stoffel EM, Brand RE, Goggins M (2023). Pancreatic Cancer: changing Epidemiology and New approaches to Risk Assessment, early detection, and Prevention. Gastroenterology.

[5] Klein AP (2021). Pancreatic cancer epidemiology: understanding the role of lifestyle and inherited risk factors. Nat Rev Gastroenterol Hepatol.

[6] Tintelnot J, Xu Y, Lesker TR, Schönlein M, Konczalla L, Giannou AD (2023). Microbiota-derived 3-IAA influences chemotherapy efficacy in pancreatic cancer. Nature.

[7] Halbrook CJ, Lyssiotis CA, Pasca di Magliano M, Maitra A (2023). Pancreatic cancer: advances and challenges. Cell.

[8] Kartal E, Schmidt TSB, Molina-Montes E, Rodríguez-Perales S, Wirbel J, Maistrenko OM (2022). A faecal microbiota signature with high specificity for pancreatic cancer. Gut.

[9] Chen Y, Yang S, Tavormina J, Tampe D, Zeisberg M, Wang H (2022). Oncogenic collagen I homotrimers from cancer cells bind to α3β1 integrin and impact tumor microbiome and immunity to promote pancreatic cancer. Cancer Cell.

[10] Riquelme E, Zhang Y, Zhang L, Montiel M, Zoltan M, Dong W (2019). Tumor Microbiome Diversity and Composition Influence Pancreatic Cancer outcomes. Cell.

[11] Udayasuryan B, Ahmad RN, Nguyen TTD, Umaña A, Monét Roberts L, Sobol P (2022). Fusobacterium nucleatum induces proliferation and migration in pancreatic cancer cells through host autocrine and paracrine signaling. Sci Signal.

[12] Nagata N, Nishijima S, Kojima Y, Hisada Y, Imbe K, Miyoshi-Akiyama T (2022). Metagenomic identification of Microbial signatures Predicting Pancreatic Cancer from a multinational study. Gastroenterology.

[13] Yu Q, Newsome RC, Beveridge M, Hernandez MC, Gharaibeh RZ, Jobin C (2022). Intestinal microbiota modulates pancreatic carcinogenesis through intratumoral natural killer cells. Gut Microbes.

[14] Zhou W, Zhang D, Li Z, Jiang H, Li J, Ren R (2021). The fecal microbiota of patients with pancreatic ductal adenocarcinoma and autoimmune pancreatitis characterized by metagenomic sequencing. J Transl Med.

[15] Maekawa T, Fukaya R, Takamatsu S, Itoyama S, Fukuoka T, Yamada M (2018). Possible involvement of Enterococcus infection in the pathogenesis of chronic pancreatitis and cancer. Biochem Biophys Res Commun.

[16] Kohi S, Macgregor-Das A, Dbouk M, Yoshida T, Chuidian M, Abe T (2022). Alterations in the duodenal fluid microbiome of patients with pancreatic Cancer. Clin Gastroenterol Hepatol.

[17] Farrell JJ, Zhang L, Zhou H, Chia D, Elashoff D, Akin D (2012). Variations of oral microbiota are associated with pancreatic diseases including pancreatic cancer. Gut.

[18] Nadeem SO, Jajja MR, Maxwell DW, Pouch SM, Sarmiento JM (2021). Neoadjuvant chemotherapy for pancreatic cancer and changes in the biliary microbiome. Am J Surg.

[19] Segata N, Izard J, Waldron L, Gevers D, Miropolsky L, Garrett WS (2011). Metagenomic biomarker discovery and explanation. Genome Biol.

[20] Langille MG, Zaneveld J, Caporaso JG, McDonald D, Knights D, Reyes JA (2013). Predictive functional profiling of microbial communities using 16S rRNA marker gene sequences. Nat Biotechnol.

[21] Wang N, Fang JY (2023). Fusobacterium nucleatum, a key pathogenic factor and microbial biomarker for colorectal cancer. Trends Microbiol.

[22] Dominguez-Bello MG, Godoy-Vitorino F, Knight R, Blaser MJ (2019). Role of the microbiome in human development. Gut.

[23] Langheinrich M, Wirtz S, Kneis B, Gittler MM, Tyc O, Schierwagen R, et al. Microbiome patterns in matched bile, duodenal, pancreatic tumor tissue, drainage, and Stool Samples: Association with Preoperative Stenting and postoperative pancreatic Fistula Development. J Clin Med. 2020;9(9). 10.3390/jcm9092785. 10.3390/jcm9092785 PMC7563524 32872220

[24] Hou K, Wu ZX, Chen XY, Wang JQ, Zhang D, Xiao C (2022). Microbiota in health and diseases. Signal Transduct Target Ther.

[25] Donia MS, Fischbach MA, HUMAN MICROBIOTA (2015). Small molecules from the human microbiota. Science.

[26] Jiang X, Ma Y, Wang T, Zhou H, Wang K, Shi W (2023). Targeting UBE2T potentiates Gemcitabine Efficacy in Pancreatic Cancer by regulating pyrimidine metabolism and replication stress. Gastroenterology.

[27] Yang Y, Weng W, Peng J, Hong L, Yang L, Toiyama Y (2017). Fusobacterium nucleatum increases proliferation of Colorectal Cancer cells and Tumor Development in mice by activating toll-like receptor 4 signaling to Nuclear Factor-κB, and Up-regulating expression of MicroRNA-21. Gastroenterology.

[28] Kim JM, Eckmann L, Savidge TC, Lowe DC, Witthöft T, Kagnoff MF (1998). Apoptosis of human intestinal epithelial cells after bacterial invasion. J Clin Invest.

[29] Kennedy KM, de Goffau MC, Perez-Muñoz ME, Arrieta MC, Bäckhed F, Bork P (2023). Questioning the fetal microbiome illustrates pitfalls of low-biomass microbial studies. Nature.

[30] Olomu IN, Pena-Cortes LC, Long RA, Vyas A, Krichevskiy O, Luellwitz R (2020). Elimination of kitome and splashome contamination results in lack of detection of a unique placental microbiome. BMC Microbiol.

[31] Stevens DA, Kim KK, Johnson N, Lee JS, Hamilton JR (2013). Halomonas johnsoniae: review of a medically underappreciated genus of growing human importance. Am J Med Sci.

[32] Chao H, Sun M, Ye M, Zheng X, Hu F (2020). World within world: intestinal bacteria combining physiological parameters to investigate the response of Metaphire guillelmi to tetracycline stress. Environ Pollut.

[33] Li Y, Zhang SX, Yin XF, Zhang MX, Qiao J, Xin XH (2021). The gut microbiota and its relevance to Peripheral lymphocyte subpopulations and cytokines in patients with rheumatoid arthritis. J Immunol Res.

[34] Avilés-Jiménez F, Guitron A, Segura-López F, Méndez-Tenorio A, Iwai S, Hernández-Guerrero A (2016). Microbiota studies in the bile duct strongly suggest a role for Helicobacter pylori in extrahepatic cholangiocarcinoma. Clin Microbiol Infect.

[35] Najafi S, Abedini F, Azimzadeh Jamalkandi S, Shariati P, Ahmadi A, Gholami Fesharaki M (2021). The composition of lung microbiome in lung cancer: a systematic review and meta-analysis. BMC Microbiol.

[36] Yan R, Guo Y, Gong Q, Chen M, Guo Y, Yang P (2020). Microbiological evidences for gastric cardiac microflora dysbiosis inducing the progression of inflammation. J Gastroenterol Hepatol.

[37] Feng R, Zhang T, Kayani MUR, Wang Z, Shen Y, Su KL (2022). Patients with primary and secondary bile Duct stones Harbor distinct biliary microbial composition and metabolic potential. Front Cell Infect Microbiol.

[38] Saab M, Mestivier D, Sohrabi M, Rodriguez C, Khonsari MR, Faraji A (2021). Characterization of biliary microbiota dysbiosis in extrahepatic cholangiocarcinoma. PLoS ONE.

[39] Miyabe K, Chandrasekhara V, Wongjarupong N, Chen J, Yang L, Johnson S, et al. Potential role of inflammation-promoting biliary microbiome in primary sclerosing Cholangitis and Cholangiocarcinoma. Cancers (Basel). 2022;14(9). 10.3390/cancers14092120. 10.3390/cancers14092120 PMC9104786 35565248

