# Supplementary material for: Alterations of the bile microbiome is associated with progression-free survival in pancreatic ductal adenocarcinoma patients
Source: BMC Microbiol. 2024 Jul 1;24:235. doi: 10.1186/s12866-024-03371-9 (PMC11218221; doi:10.1186/s12866-024-03371-9)
Supplement: Supplementary file 2 — Supplementary Material 2 [file 12866_2024_3371_MOESM2_ESM.docx]

**Supplementary Figure**

**
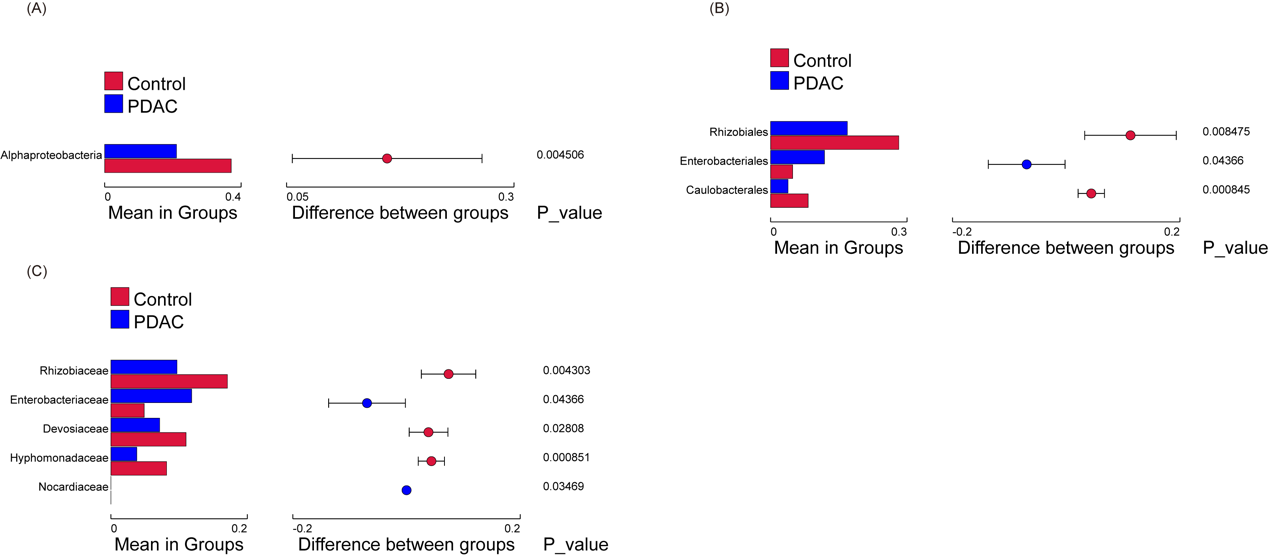
**

**Figure S1** Identification of differential bacteria between PDAC and Control groups (A) Significantly different class between two groups. (B) Significantly different orders between two groups. (C) Significantly different families between two groups.

**
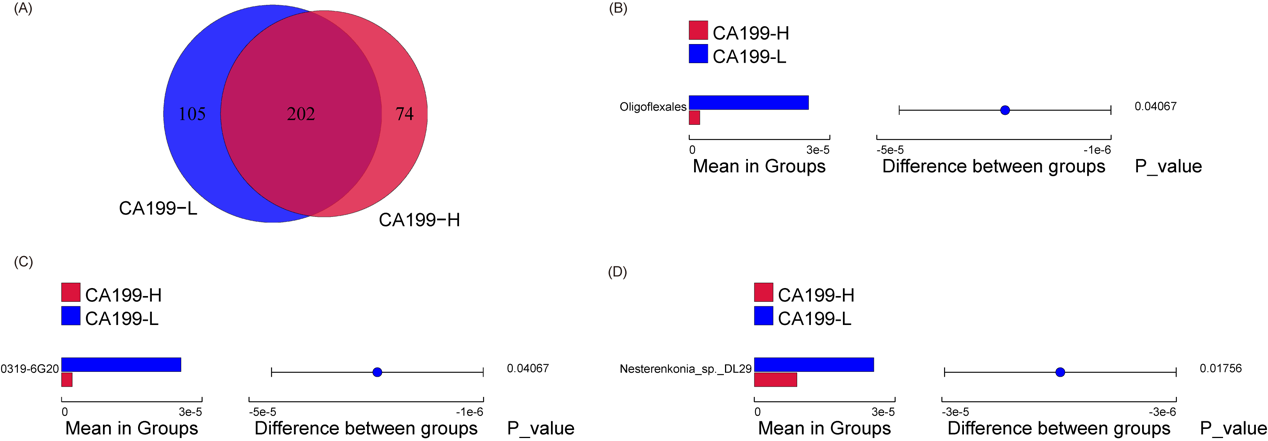
**

**Figure S2** Identification of differential bacteria between CA199-L and CA199-H groups (A) Venn diagram of the overlaps between the composition of bile microbiota based on OTUs. (B) Significantly different orders between two groups. (C) Significantly different families between two groups. (D) Significantly different species between two groups.

**
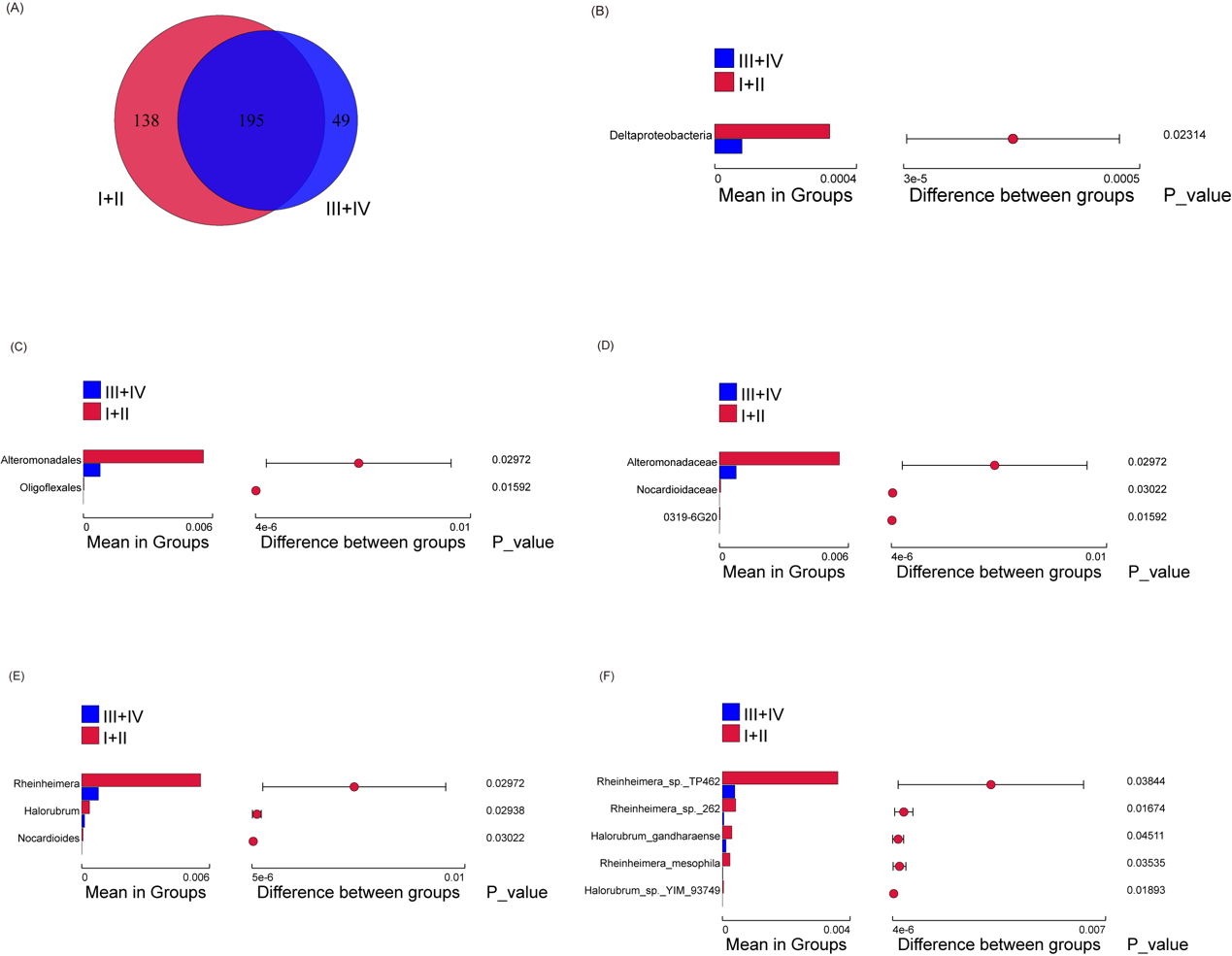
**

**Figure S3** Identification of differential bacteria between I+II and III+IV groups (A) Venn diagram of the overlaps between the composition of bile microbiota based on OTUs. Significantly different classes (B), orders (C), families (D), genera (E), species (F) between two groups.

**
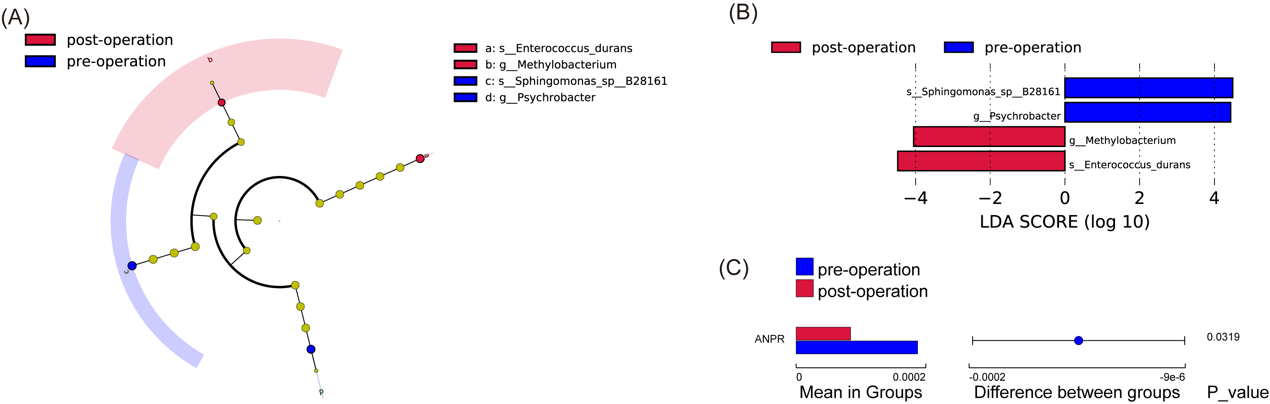
 Figure S4** Identification of underlying bile microbial biomarkers and microbial community function between post and pre-operation groups (A) (B). Significantly different genera between two groups (C).

**
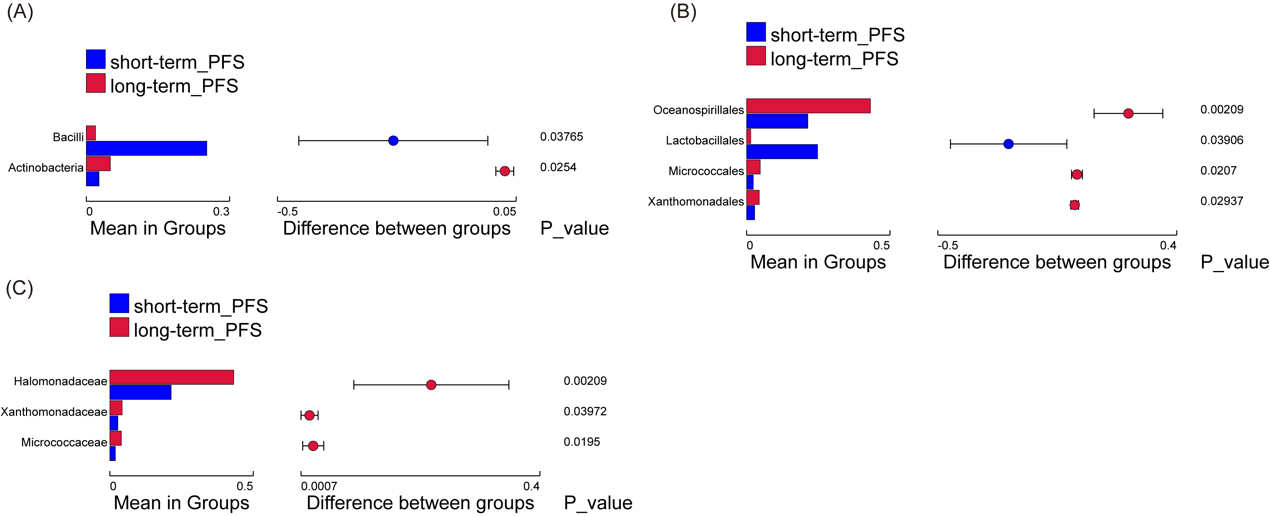
**

**Figure S5** Identification of differential bacteria between long-term PFS and short-term PFS groups Significantly different classes (A), orders (B), families (C), between two groups.

**
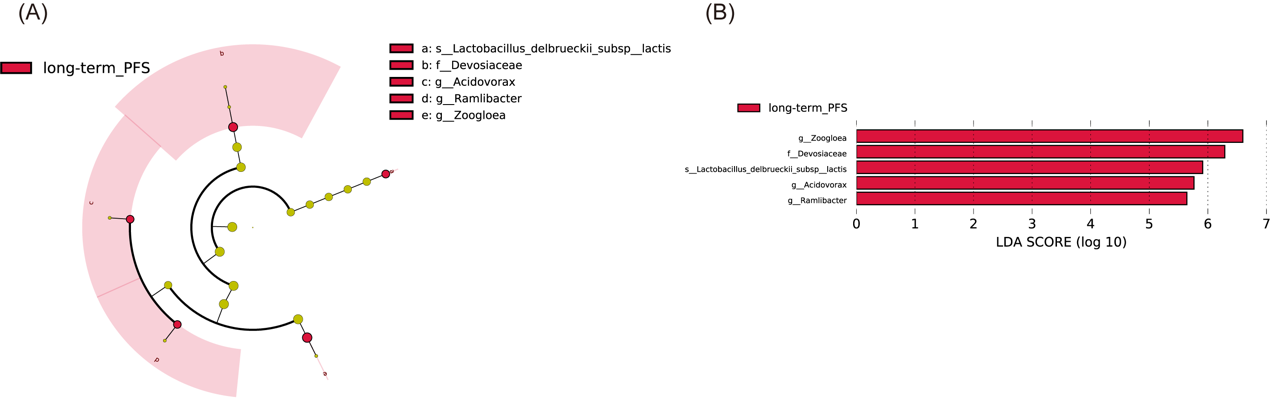
**

**Figure S6** Identification of underlying bile microbial biomarkers between long-term PFS and short-term PFS groups based on LEfSe (A) (B).
